# Mammalian SWI/SNF Enzymes and the Epigenetics of Tumor Cell Metabolic Reprogramming

**DOI:** 10.3389/fonc.2017.00049

**Published:** 2017-04-04

**Authors:** Jeffrey A. Nickerson, Qiong Wu, Anthony N. Imbalzano

**Affiliations:** ^1^Department of Cell and Developmental Biology, University of Massachusetts Medical School, Worcester, MA, USA; ^2^Department of Pediatrics, University of Massachusetts Medical School, Worcester, MA, USA; ^3^Department of Biochemistry and Molecular Pharmacology, University of Massachusetts Medical School, Worcester, MA, USA

**Keywords:** SMARCA4, breast cancer, SWI/SNF, fatty acid synthesis pathway, chromatin remodeling, epigenetic regulation, cancer metabolism

## Abstract

Tumor cells reprogram their metabolism to survive and grow in a challenging microenvironment. Some of this reprogramming is performed by epigenetic mechanisms. Epigenetics is in turn affected by metabolism; chromatin modifying enzymes are dependent on substrates that are also key metabolic intermediates. We have shown that the chromatin remodeling enzyme Brahma-related gene 1 (BRG1), an epigenetic regulator, is necessary for rapid breast cancer cell proliferation. The mechanism for this requirement is the BRG1-dependent transcription of key lipogenic enzymes and regulators. Reduction in lipid synthesis lowers proliferation rates, which can be restored by palmitate supplementation. This work has established BRG1 as an attractive target for breast cancer therapy. Unlike genetic alterations, epigenetic mechanisms are reversible, promising gentler therapies without permanent off-target effects at distant sites.

Tumor cells reprogram their metabolism to support growth in their unique and challenging microenvironment, a hypoxic environment with inadequate blood supply for normal nutrient replenishment. As first observed by Otto Warburg (1, 2), tumor cells develop a glycolytic metabolism where energy is derived primarily from nutrient catabolism to lactate and not from the mitochondrial Krebs cycle where cells in normal tissue derive most of their energy. Although adaptive to a hypoxic tumor microenvironment, this preference for glycolysis persists even when oxygen is abundant. The nutrient fuel for glycolysis is glucose, but tumor cells also become “addicted” to the normally non-essential amino acid glutamine (3), as first observed in cultured cells by Harry Eagle (4). Glutamine can serve as a carbon and nitrogen source for amino acid synthesis and can fuel the residual Krebs cycle after conversion to glutamate and then α-ketoglutarate. After a period of neglect, cancer metabolism is now recognized as central to the cancer phenotype and as an important target for the development of therapies (5).

## Regulation of Metabolism

Cells carefully regulate their metabolism with nested levels of controls (6). First, levels of circulating molecules that serve as feedstock for metabolic pathways change with diet. These include plasma-free fatty acids and amino acids that increase after a meal (7) or plasma ketone bodies and free fatty acids that increase after a prolonged fast (8, 9). Second, allosteric regulation of metabolic enzymes changes flux rates through metabolic pathways in response to concentrations of substrates or products (10–12). Third, there is regulation by hormones (13), often through posttranslational modification of metabolic enzymes. For example, glycogen deposition or depletion is regulated by a protein kinase cascade-modifying glycogen synthase and glycogen phosphorylase downstream of insulin or glucagon (14, 15). AMP-activated Kinase (AMPK) is a master regulator of metabolism that can sense cellular energy status and respond by switching on and off pathways to achieve energy homeostasis (16, 17). AMPK is activated in response to cellular ATP depletion, which can result from low glucose levels, hypoxia, and heat shock. Upon activation, AMPK upregulates pathways replenishing ATP, including fatty acid β-oxidation and autophagy, and downregulates ATP-consuming processes, including lipid synthesis and protein synthesis.

The protein kinase mTOR (mechanistic target of rapamycin) (18, 19) is the core Ser/Thr protein kinase in two signal transduction complexes, mTORC1 and mTORC2. mTORC1 is a master growth regulator that senses and integrates diverse signals, including levels of growth factors, amino acids, other metabolites, and cellular stress. mTORC2 activates the cell signaling Ser/Thr protein kinase AKT, promotes cellular survival, regulates cytoskeletal dynamics, and regulates growth *via* SGK1 phosphorylation. mTOR complexes promote cell growth through regulation of anabolic and catabolic metabolic processes by multiple mechanisms, as well as through control of cell proliferation. An altered interplay of all of these mechanisms participates in the progressive reprogramming of metabolism with tumor progression.

## Transcriptional Regulation of Metabolism

Metabolic pathways can also be regulated by transcriptional mechanisms increasing or decreasing levels of enzymes. Take the example of lipid metabolism. The Sterol Regulatory Element Binding Protein (SREBP) transcription factors are the master regulators that control the expression of nearly all lipogenic enzymes. The mTORC1 complex regulates lipid synthesis (20) through SREBP by multiple mechanisms. In response to cellular signaling, mTORC1 regulates SREBP processing through S6K and increases SREBP nuclear accumulation through Lipin 1, a phosphatidic acid phosphatase that is also a transcriptional coactivator (21–25). mTORC1 phosphorylates Lipin1, preventing its translocation into the nucleus where it can inhibit SREBP1/2-dependent transcription (24). mTORC1 also increases the activity and expression of peroxisome proliferator-activated receptor γ (PPARγ), another transcriptional regulator of lipogenic genes (26, 27). By these mechanisms, mTORC1 increases the transcription of lipogenic genes, including key enzymes in fatty acid synthesis, such as acetyl CoA carboxylase (ACC), ATP citrate lyase (ACLY), and fatty acid synthase (FASN). As we shall discuss, we have shown Lipin1 and each of these enzymes involved in fatty acid synthesis to be transcriptionally regulated by Brahma-related gene 1 (BRG1), a chromatin remodeling enzyme (28).

## Cancer Epigenetics

Epigenetic mechanisms control heritable phenotypes without changes in DNA sequence, often changing chromatin structure by modulating DNA methylation, the posttranslational modification of histones and non-histone chromatin associated proteins, and the regulation of ATP-dependent chromatin remodeling enzymes that control genome accessibility (29). Epigenetic mechanisms regulate normal development and maintain tissue-specific gene expression patterns while their disruption can cause altered gene function and contribute to malignant cellular transformation. The initiation and progression of cancer has been seen as a genetic disease, but we now realize that epigenetic abnormalities contribute to the development of cancer. Cancer cells often have altered levels or activities of epigenetic regulatory proteins with consequences including altered chromatin structure and altered regulation of gene expression (30, 31). These are so common and numerous that global changes in the epigenetic landscape are now considered a hallmark of cancer (5).

## The Role of BRG1 in Cancer Epigenetics is Context Dependent

Chromatin structure presents a barrier to transcription factors and polymerases accessing DNA. Several multiprotein complexes alter chromatin structure using the energy derived from ATP-hydrolysis (32–34), including the mammalian SWI/SNF family of chromatin modifiers, which are large, multisubunit enzymes that contain one of two closely related ATPases called BRM or BRG1 (35–37). SWI/SNF complexes containing either catalytic subunit alter nucleosome structure and facilitate binding of transcription factors to nucleosomal DNA in an ATP-dependent manner (38, 39). Subunits of the mammalian SWI/SNF complexes are important for gene activation and repression, development and differentiation, recombination and repair, cell cycle control, and tumorigenesis (40–43). For example, the SNF5 (INI1) subunit is required for embryonic development and functions as a tumor suppressor (44–46).

Brahma-related gene 1 (BRG1) function in cancer is context dependent. BRG1 is mutated in lung and other cancers, where it may function as a tumor suppressor (30, 47). Cancers that have lost the SWI/SNF INI1 subunit require BRG1 (48), suggesting that targeting BRG1 may be therapeutic for these tumors. Similarly, targeting BRM might be an effective strategy for targeting BRG1-deficient tumors (49, 50). As we and others have shown, BRG1 is upregulated but rarely mutated in primary breast and prostate tumors, in melanoma and neuroblastoma, and in pancreatic, gastric, and colorectal carcinomas (51–60). Mice heterozygous for Brg1 develop mammary tumors (61, 62). However, conditional knockout of Brg1 in mammary gland does not cause mammary tumors (63). Genome sequencing of more than 500 primary breast cancers showed none with mutations in BRG1 (64). The evidence suggests that BRG1 can be a driver of cancer as well as a tumor suppressor.

## Fatty Acid Metabolism and Cancer

In tissues with high rates of lipogenesis such as liver, lactating mammary gland, and adipose tissue, the fatty acid synthesis pathway has three principal functions: storage of excess energy as fat, synthesis of lipids from carbohydrate or protein precursors when dietary lipids are scarce, and synthesis of milk fats during lactation. Most normal cells in other tissues do not synthesize fatty acids *de novo* but preferentially use circulating lipids. However, upregulation of both lipogenic genes and overall lipogenesis are observed widely in tumors in those non-lipogenic tissues (65). Depending on the tumor type, tumor cells synthesize up to 95% of saturated and mono-unsaturated fatty acids *de novo* from acetyl CoA despite a sufficient exogenous supply of fatty acids (66). Lipogenic enzymes, such as FASN, ACC, and ACLY that are required for fatty acid biosynthesis, and SREBP1, the master regulator of lipogenic gene expression, are overexpressed in many cancers, including breast (67–70). FASN is a key enzyme involved in energy storage from excess carbohydrates to fat in liver and adipose tissue, during lactation in breast, and in support of reproduction in endometrium and decidua. But FASN expression during these processes is strictly regulated by nutrition and hormonal levels. In contrast, FASN is highly expressed in many cancer and precancerous lesions. The expression of FASN is independent of nutrition, in many cancers, as well as independent of hormonal regulation. Whereas various tumor types have elevated endogenous fatty acid biosynthesis irrespective of extracellular lipid availability, most normal cells, even those proliferating rapidly, preferentially use exogenous lipids for synthesis of new structural lipids (65, 71).

The activation of the *de novo* fatty acid synthesis pathway is not only observed in tumors but also may be required for malignant progression (65, 72, 73). For example, elevated levels of FASN, the enzyme catalyzing the synthesis of palmitate and thereby required for long chain and unsaturated fatty acid synthesis, are correlated with poor prognosis in breast cancer patients (65, 72). Increases in FASN activity and expression are observed early in cancer development and correlate with cancer progression, while high FASN levels correlate with more aggressive malignant phenotypes (65). Inhibiting key enzymes involved in fatty acid synthesis, including FASN, ACC, and ACLY, with small molecules or knockdowns reduces cell proliferation, induces the apoptosis of cancer cells, and decreases the growth of human tumors grown as mouse xenografts (65, 71, 74–77).

## BRG1 is Necessary for Fatty Acid Biosynthesis in Support of Proliferation in Breast Cancer

We first reported that the alternative SWI/SNF chromatin remodeling enzyme ATPases BRG1 and BRM are required for proliferation of breast cancer cells (59). Western blots of biopsies showed that BRG1 protein levels were higher in tumor than in normal tissue. Analysis of TCGA Breast Cancer patient data revealed an approximate twofold increase in BRG1 mRNA levels (64) and in BRG1 protein levels (78) in tumors compared to normal tissue across all subtypes. These are not well-controlled comparisons because of the great heterogeneity in normal tissue cell types. More convincingly, immunohistochemistry confirmed that the BRG1 and BRM proteins are greatly overexpressed in most primary breast cancers independent of receptor status (55, 59). BRG1 staining was rarely observed in the normal ductal epithelial cells from which most breast tumors derive but was seen in normal myoepithelial cells. However, in tumors BRG1 staining was observed in almost every cell. Because of the heterogeneity of breast cancer subtypes our further experimental work focused on triple-negative breast cancer, the most aggressive and deadly type.

Knockdown of either ATPase in triple-negative breast cancer cell lines reduced cell proliferation *in vitro* and tumor formation in xenografts. An extended cell cycle progression time was observed without apoptosis, without senescence, or without alterations in migration or attachment. Combined knockdown of BRM and BRG1 produced additive effects, suggesting that these enzymes function, at least in part, through independent mechanisms. Knockout of BRG1 or BRM using CRISPR/Cas9 technology caused cell death. Our work supports the novel idea that overexpression of BRG1 and BRM is common in breast cancer and that BRG1 and BRM are required for breast cancer cell proliferation and survival. These results are in direct contrast to other tumors where BRG1 acts as a tumor suppressor (79). For example, it is mutated in lung and other cancers. We and others have now shown that BRG1 is upregulated but rarely mutated in primary breast and prostate tumors, in melanoma and neuroblastoma, and in pancreatic, gastric, and colorectal carcinomas (51–60, 80).

When we began our studies, it was expected that BRG1 was a weak tumor suppressor in mammary gland because about 10% of Brg1+/− mice eventually developed mammary tumors (61, 62) and because there were functional interactions between BRG1 and cell cycle regulatory proteins, including RB and p53 (30, 42, 81). This tentative identification of BRG1 as a mammary tumor suppressor was challenged by our work (59) and by others (55). The conditional knockout of Brg1 in the mouse mammary gland did not cause mammary tumors (63). We observed that fewer than 2% of BRG1 sequences in the TCGA database contained mutations. Breast cancer is not alone in this requirement for BRG1. BRG1 is also required for the proliferation of HeLa cells and mouse fibroblasts (82, 83).

**Figure 1:**
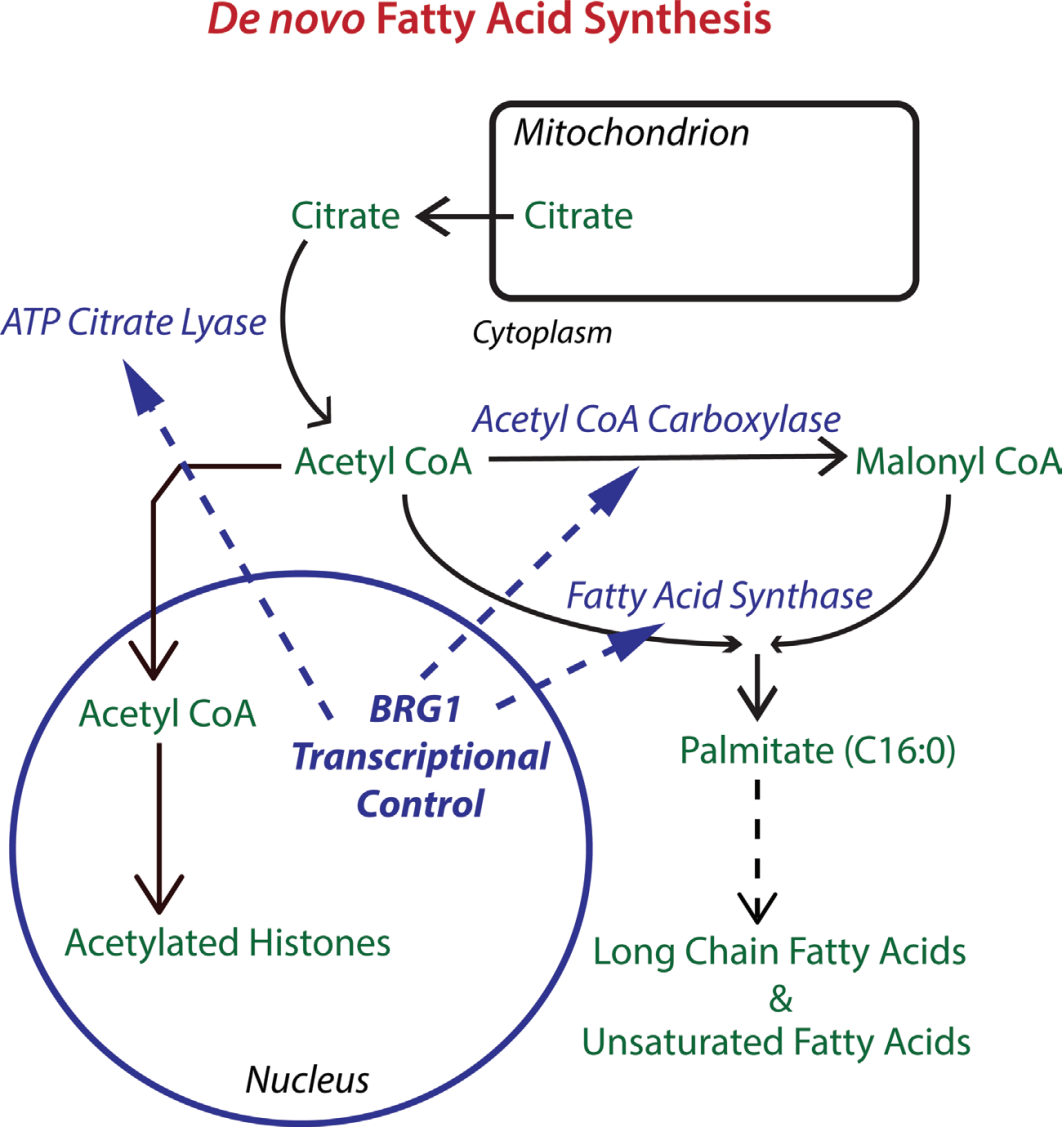
**The chromatin remodeling enzyme Brahma-related gene 1 (BRG1) epigenetically regulates key enzymes in *de novo* fatty acid biosynthesis**. The pathway for *de novo* fatty acid synthesis requires the enzymes ATP citrate lyase (ACLY), acetyl CoA carboxylase (ACC), and fatty acid synthase (FASN). ACLY is important for increasing cytoplasmic acetyl CoA to levels supportive of fatty acid synthesis. ACC is required for making malonyl CoA, which along with acetyl CoA is used by FASN to produce palmitate, a 16-carbon saturated fatty acid that can be extended and desaturated into the extended family of fatty acids which are used for fat storage and for the biosynthesis of membrane phospholipids. BRG1 is important for the transcription of ACLY, ACC, and FASN in breast cancer cells. Knockdown or inhibition of BRG1 decreases levels of all three enzymes with resulting decreases in lipid synthesis and decreases in breast tumor cell proliferation. Proliferation can be rescued with palmitate supplementation (28). Acetyl CoA is also the source of acetyl groups for histone acetylation which generally upregulates transcription and may cooperate with BRG1 in the regulation of gene expression.

What is the mechanism for the BRG1 requirement for breast cancer cell proliferation? We discovered that BRG1 promotes breast cancer by reprogramming lipid synthesis (28) as shown in Figure 1. BRG1 knockdown reduced the rate of chloroform/methanol extractable lipid synthesis by 35% while glucose uptake remained unchanged. mRNA and protein levels for ACC, ACLY, and FASN, the key enzymes in *de novo* fatty acid synthesis, were all significantly decreased in BRG1 knockdown cells as were other important proteins performing or regulating lipid synthesis such as Lipin1. BRG1 bound to the promoters of all of these genes, and the promoter binding was diminished in BRG1 knockdown cells, evidence of direct BRG1 transcriptional control. Treatment with either an ACC inhibitor or a FASN inhibitor decreased cell number, and BRG1 knockdown cells showed increased sensitivity to these inhibitors. Remarkably, addition of exogenous palmitate, the key intermediate in fatty acid synthesis, completely rescued proliferation. Our work supports a mechanism in which BRG1 transcriptionally promotes *de novo* lipid synthesis, which is necessary for maintaining high rates of proliferation. In these cells, exogenous palmitate can substitute for endogenous FASN-generated palmitate. Furthermore, BRG1 regulation of proliferation through fatty acid metabolism is breast cancer specific. We showed that key fatty acid synthesis enzymes are not upregulated by BRG1 in non-tumorigenic MCF-10A mammary epithelial cells (59). Though MCF-10A cells also require BRG1 for proliferation (84), this requirement has a different mechanism. Restoration of BRG1 expression in cells depleted for both BRG1 and BRM rescued lipid synthesis, the expression of lipogenic enzymes and cell proliferation so BRM is not required for these effects in this system.

## Targeting BRG1 for Breast Cancer Therapy

Chromatin remodeling complexes have not been viewed as a drugable target until recently, but our work shows that the BRG1 chromatin remodeling enzyme is an especially promising target for epigenetic breast cancer chemotherapy (28, 59, 85). Inhibition of BRG1 function decreases tumor cell proliferation, decreases tumor mass in mouse models, and potentiates tumor cell killing by clinically used chemotherapy drugs.

Only two BRG1 inhibitors have been reported. PFI-3, a Pfizer/Structural Genomics Consortium candidate, is a small molecule inhibitor that specifically targets the bromo domains of BRG1, BRM, and PB1 (86, 87). We treated three triple-negative breast cancer cell lines, MDA-MB-231, MDA-MB-468, and HDQ-P1, with PFI-3 at different doses (85). No inhibition of cell proliferation was observed. This is consistent with recent results demonstrating that PFI-3 does not affect the proliferation rate of other cancer cell lines (87). While PFI-3 does have an effect on some BRG1 functions, it does not dislodge full length BRG1 from chromatin (87) and this may be necessary for inhibiting proliferation through control of lipid synthesis.

The natural product ADAADi (Active DNA-dependent ATPase A Domain inhibitor) inhibits the ATPase activity of the SWI2/SNF2 family of ATPases (88, 89). Enzymes from other families of DNA-dependent ATPases have no or greatly reduced sensitivity to ADAADi, and DNA-independent or RNA-dependent ATPases are not affected (88). ADAADi inhibits BRG1 nucleosome remodeling activity *in vitro* (88). We tested the ADAADi inhibitor on TNBC cell lines: MDA-MB-231, MDA-MB-468, and HDQ-P1. ADAADiN significantly decreased cell proliferation in these cell lines (85). However, ADAADi failed to decrease cell proliferation significantly in cells with experimentally reduced BRG1 expression. This observation strongly suggests that ADAADiN specifically targeted BRG1 in these cells by interfering with its ATPase function.

ADAADi decreases lipid biosynthesis in breast cancer cells (28) and also sensitizes cells to chemotherapy drugs, just as BRG1 knockdown does (85). After pretreatment with ADAADi, cells were exposed to different doses of six clinically used chemotherapy drugs and cell viability was assayed by MTT. ADAADi significantly increased the drug killing efficacy in MDA-MB-231 and MDA-MB-468 cells from 3-fold to over 10-fold. Therefore, chemical inhibition of the BRG1 ATPase domain targets BRG1-mediated pro-survival pathways in breast cancer cells, decreasing levels of the ABC transporters that pump chemotherapy drugs out of cells and contribute to treatment failure (85).

## Metaboloepigenetics

At the level of organisms, food intake affects patterns of gene expression. At the level of cells, levels of nutrients and metabolites regulate patterns of gene expression. Multiple mechanisms have been described and many remain to be discovered (90–92). Epigenetic controls are often exerted through covalent modifications of chromatin proteins or through modification of DNA itself. The essential donor groups for these modifications are important metabolic intermediates including Acetyl CoA, S-adenosylmethionine, ATP, and NAD+.

Here, we will concentrate on histone acetylation and metabolism. In one form of epigenetic regulation, histones can be acetylated at multiple positions on their N-terminal tail domains, affecting gene expression at the proximate genes. The extent of histone acetylation at specific sites depends on relative rates of deposition by histone acetyl transferases and removal by Histone Deacetylases (HDACs). The acetyl donor for histone acetylases is acetyl CoA, a metabolite that is produced downstream of glycolysis by the mitochondrial trichloroacetic acid cycle, by the β-oxidation of fatty acids, or by amino acid catabolism. Acetyl CoA is required for both fatty acid and cholesterol synthesis. ACLY generates acetyl CoA from citrate, ATP, and CoA (Figure 1). It partitions to both nucleus and cytoplasm, suggesting that nuclear acetyl CoA can be made locally (93) and that nucleocytoplasmic levels change with the metabolic status of cells, for example with glucose levels (94, 95). Knocking down ACLY reduces the acetylation of core histones H2B, H3, and H4 with consequent reductions in the expression proximate genes (93). As we have found ACLY to be transcriptionally regulated by the chromatin remodeling enzyme BRG1 in triple-negative breast cancer cells (28), BRG1-mediated chromatin remodeling may tune the relationship between metabolism and histone acetylation, linking two distinct mechanisms for epigenetic regulation.

Histones can also be acylated with at least eight other species of short chain carboxyl groups: propionyl, butyryl, 2-hydroxyisobutyryl, succinyl, malonyl, glutaryl, crotonyl and β-hydroxybutyryl (94, 96). The levels of these modifications may be controlled by the metabolic pathways producing these carboxyl groups. This may be a mechanism for integrating readouts from these pathways to control patterns of transcription. There is now evidence that histones are acylated with longer chain fatty acids (97). Such a mechanism would directly link fatty acid levels with histone epigenetics.

Many HDACs exist in mammalian cells. Class III HDACs, also known as sirtuins, are nicotinamide adenine dinucleotide (NAD)-dependent deacetylases (98). NAD is a coenzyme carrying electrons between redox reactions in its reduced form NADH. More than 200 metabolic enzymes use NAD+/NADH as a cofactor, most functioning in catabolism. For example, starting with one glucose molecule, two NAD+ molecules are reduced to NADH in glycolysis, at the step catalyzed by glyceraldehyde 3-phosphate dehydrogenase. The highly related NADP+/NADPH performs the same role for enzyme catalyzed anabolic reactions, for example in the *de novo* synthesis of palmitate by FASN.

As first shown for SIRT2 (99–101), a cytoplasmically localized protein, sirtuins have a deacetylation activity requiring NAD+, but not as an electron carrier. Instead, their reactions use NAD+ in equal stoichiometry to the acetyl group and cleave NAD, generating nicotinamide and 2′-*O*-acetyl-ADP-ribose. Of the seven mammalian sirtuins, SIRT1, SIRT 6, and SIRT 7 are nuclear proteins, enriched in the nucleoplasm, in heterochromatin, and in nucleoli, respectively, and positioned to deacetylate histones and other nuclear proteins (102). SIRT1 efficiently deacetylates p53 (102).

It has been proposed that this unusual use of NAD+ makes these sirtuins sensors of cellular NAD+ levels. Cellular and nuclear NAD+ levels are close enough to the Km of SIRT1 for NAD+ to make this plausible (103). In this view, cellular NAD+ levels would change in response to metabolic fluxes or stresses and cause changes in histone and other nuclear protein acetylation with consequences on gene expression. Conflicts have been noted between this model and early studies on NAD+ levels that showed little response to starvation (92). NAD+ levels do cycle with circadian rhythms (104) and increase with exercise (105). NAD+/NADH ratios decrease in response to elevated glucose levels in C2C12 skeletal muscle cells while in the muscles of fasted mice SIRT1 decreases expression of AMPK targets in control animals and is necessary for their induction after fasting (106). In mouse liver, NAD+ levels are increased by 33% after fasting for 24 h and return to control levels after 24 h after refeeding (107). SIRT1 protein levels were induced after refeeding, showing a second mechanism for SIRT1 activity regulation. The energy sensor AMPK increases cellular NAD+ levels, increasing SIRT1 deacetylation of downstream SIRT1 targets (108). SIRT1 is proposed to activate AMPK creating a feedback loop between SIRT1 and AMPK that controls energy metabolism.

## Therapeutic Intervention in Breast Cancer Epigenetics and Metabolism

The reciprocal relationships between metabolism and epigenetic regulation are attractive opportunities for targeted cancer therapy. Multiple drug candidates targeting epigenetic mechanisms are currently in trials for breast cancer. Among those with published promising results are the HDAC inhibitors SAHA (Vorinostat) (109–111), entinostat (112), valproate (113), and romidepsin (114). Romidepsin and vorinostat have been FDA approved for treatment of T-cell lymphomas (115, 116). An inhibitor of a DNA methyltransferase, 5-aza-2′-deoxycytidine (5azaC), causes DNA hypomethylation, is FDA approved for treatment of myelodysplastic syndrome (117, 118), and has early promise for breast cancer (119, 120). The great promise of these drugs should drive the search for other epigenetic targets in cancer therapy.

In the work, we have reviewed here, the chromatin remodeling enzyme BRG1 and its breast cancer-specific effects on lipid metabolism are an attractive target for breast cancer therapy. Our work establishes that one part of the anti-cancer mechanism of BRG1-targeted drugs is an effect on fatty acid synthesis decreasing proliferation. Unlike genetic alterations, epigenetic mechanisms are reversible, promising gentler therapies without permanent off-target effects at distant sites.

## Author Contributions

JN wrote sections and edited the contributions of the other authors. AI and QW wrote sections and edited the manuscript. All three authors contributed to the published work reviewed in this article.

## Conflict of Interest Statement

The authors declare that the research was conducted in the absence of any commercial or financial relationships that could be construed as a potential conflict of interest.

## References

[1] Warburg O Posener K Negelein E Über den Stoffwechsel der Carcinomzelle. Biochem Z (1924) 152:309–44.

[2] Warburg O On the origin of cancer cells. Science (1956) 123(3191):309–14. 10.1126/science.123.3191.309 13298683

[3] Wise DR Thompson CB. Glutamine addiction: a new therapeutic target in cancer. Trends Biochem Sci (2010) 35(8):427–33. 10.1016/j.tibs.2010.05.003 20570523 PMC2917518

[4] Eagle H Nutrition needs of mammalian cells in tissue culture. Science (1955) 122(3168):501–14. 10.1126/science.122.3168.501 13255879

[5] Hanahan D Weinberg RA Hallmarks of cancer: the next generation. Cell (2011) 144(5):646–74. 10.1016/j.cell.2011.02.013 21376230

[6] Nelson DL Cox MM Lehninger Principles of Biochemistry. New York: Worth Publishers (2000).

[7] Frayn KN. Adipose tissue as a buffer for daily lipid flux. Diabetologia (2002) 45(9):1201–10. 10.1007/s00125-002-0873-y 12242452

[8] Cahill GF Jr Aoki TT Ruderman NB Ketosis. Trans Am Clin Climatol Assoc (1973) 84:184–202. 4199621 PMC2441301

[9] Cahill GF Jr. Fuel metabolism in starvation. Annu Rev Nutr (2006) 26:1–22. 10.1146/annurev.nutr.26.061505.111258 16848698

[10] Changeux JP Allosteric interactions interpreted in terms of quaternary structure. Brookhaven Symp Biol (1964) 17:232–49. 14246265

[11] Monod J. From enzymatic adaptation to allosteric transitions. Science (1966) 154(3748):475–83. 10.1126/science.154.3748.475 5331094

[12] Stadtman ER Allosteric regulation of enzyme activity. Adv Enzymol Relat Areas Mol Biol (1966) 28:41–154. 5334065 10.1002/9780470122730.ch2

[13] Randle PJ Endocrine control of metabolism. Annu Rev Physiol (1963) 25:291–324. 10.1146/annurev.ph.25.030163.001451 13990766

[14] Krebs EG Protein kinases. Curr Top Cell Regul (1972) 5:99–133. 10.1016/B978-0-12-152805-8.50010-1 4358204

[15] Hers HG. The control of glycogen metabolism in the liver. Annu Rev Biochem (1976) 45:167–89. 10.1146/annurev.bi.45.070176.001123 183599

[16] Hardie DG AMPK – sensing energy while talking to other signaling pathways. Cell Metab (2014) 20(6):939–52. 10.1016/j.cmet.2014.09.013 25448702 PMC5693325

[17] Carling D Viollet B. Beyond energy homeostasis: the expanding role of AMP-activated protein kinase in regulating metabolism. Cell Metab (2015) 21(6):799–804. 10.1016/j.cmet.2015.05.005 26039446

[18] Sabatini DM Erdjument-Bromage H Lui M Tempst P Snyder SH. RAFT1: a mammalian protein that binds to FKBP12 in a rapamycin-dependent fashion and is homologous to yeast TORs. Cell (1994) 78(1):35–43. 10.1016/0092-8674(94)90570-3 7518356

[19] Laplante M Sabatini DM. mTOR signaling in growth control and disease. Cell (2012) 149(2):274–93. 10.1016/j.cell.2012.03.017 22500797 PMC3331679

[20] Laplante M Sabatini DM. An emerging role of mTOR in lipid biosynthesis. Curr Biol (2009) 19(22):R1046–52. 10.1016/j.cub.2009.09.058 19948145 PMC3390254

[21] Porstmann T Santos CR Griffiths B Cully M Wu M Leevers S SREBP activity is regulated by mTORC1 and contributes to Akt-dependent cell growth. Cell Metab (2008) 8(3):224–36. 10.1016/j.cmet.2008.07.007 18762023 PMC2593919

[22] Duvel K Yecies JL Menon S Raman P Lipovsky AI Souza AL Activation of a metabolic gene regulatory network downstream of mTOR complex 1. Mol Cell (2010) 39(2):171–83. 10.1016/j.molcel.2010.06.022 20670887 PMC2946786

[23] Li S Brown MS Goldstein JL. Bifurcation of insulin signaling pathway in rat liver: mTORC1 required for stimulation of lipogenesis, but not inhibition of gluconeogenesis. Proc Natl Acad Sci U S A (2010) 107(8):3441–6. 10.1073/pnas.0914798107 20133650 PMC2840492

[24] Peterson TR Sengupta SS Harris TE Carmack AE Kang SA Balderas E mTOR complex 1 regulates lipin 1 localization to control the SREBP pathway. Cell (2011) 146(3):408–20. 10.1016/j.cell.2011.06.034 21816276 PMC3336367

[25] Wang BT Ducker GS Barczak AJ Barbeau R Erle DJ Shokat KM. The mammalian target of rapamycin regulates cholesterol biosynthetic gene expression and exhibits a rapamycin-resistant transcriptional profile. Proc Natl Acad Sci U S A (2011) 108(37):15201–6. 10.1073/pnas.1103746108 21876130 PMC3174577

[26] Kim JE Chen J. regulation of peroxisome proliferator-activated receptor-gamma activity by mammalian target of rapamycin and amino acids in adipogenesis. Diabetes (2004) 53(11):2748–56. 10.2337/diabetes.53.11.2748 15504954

[27] Zhang HH Huang J Duvel K Boback B Wu S Squillace RM Insulin stimulates adipogenesis through the Akt-TSC2-mTORC1 pathway. PLoS One (2009) 4(7):e6189. 10.1371/journal.pone.0006189 19593385 PMC2703782

[28] Wu Q Madany P Dobson JR Schnabl JM Sharma S Smith TC The BRG1 chromatin remodeling enzyme links cancer cell metabolism and proliferation. Oncotarget (2016) 7:38270–81. 10.18632/oncotarget.9505 27223259 PMC5122388

[29] Teperino R Lempradl A Pospisilik JA. Bridging epigenomics and complex disease: the basics. Cell Mol Life Sci (2013) 70(9):1609–21. 10.1007/s00018-013-1299-z 23463237 PMC11113658

[30] Wilson BG Roberts CW. SWI/SNF nucleosome remodellers and cancer. Nat Rev Cancer (2011) 11(7):481–92. 10.1038/nrc3068 21654818

[31] Dawson MA Kouzarides T Cancer epigenetics: from mechanism to therapy. Cell (2012) 150(1):12–27. 10.1016/j.cell.2012.06.013 22770212

[32] Bowman GD. Mechanisms of ATP-dependent nucleosome sliding. Curr Opin Struct Biol (2010) 20(1):73–81. 10.1016/j.sbi.2009.12.002 20060707 PMC2947954

[33] Flaus A Owen-Hughes T. Mechanisms for ATP-dependent chromatin remodelling: the means to the end. FEBS J (2011) 278(19):3579–95. 10.1111/j.1742-4658.2011.08281.x 21810178 PMC4162296

[34] Hota SK Bartholomew B. Diversity of operation in ATP-dependent chromatin remodelers. Biochim Biophys Acta (2011) 1809(9):476–87. 10.1016/j.bbagrm.2011.05.007 21616185 PMC3171594

[35] Khavari PA Peterson CL Tamkun JW Mendel DB Crabtree GR. BRG1 contains a conserved domain of the SWI2/SNF2 family necessary for normal mitotic growth and transcription. Nature (1993) 366(6451):170–4. 10.1038/366170a0 8232556

[36] Muchardt C Yaniv M. A human homologue of *Saccharomyces cerevisiae* SNF2/SWI2 and *Drosophila brm* genes potentiates transcriptional activation by the glucocorticoid receptor. EMBO J (1993) 12(11):4279–90. 8223438 10.1002/j.1460-2075.1993.tb06112.x PMC413724

[37] Chiba H Muramatsu M Nomoto A Kato H. Two human homologues of *Saccharomyces cerevisiae* SWI2/SNF2 and *Drosophila brahma* are transcriptional coactivators cooperating with the estrogen receptor and the retinoic acid receptor. Nucleic Acids Res (1994) 22(10):1815–20. 10.1093/nar/22.10.1815 8208605 PMC308079

[38] Imbalzano AN Kwon H Green MR Kingston RE. Facilitated binding of TATA-binding protein to nucleosomal DNA. Nature (1994) 370(6489):481–5. 10.1038/370481a0 8047170

[39] Kwon H Imbalzano AN Khavari PA Kingston RE Green MR. Nucleosome disruption and enhancement of activator binding by a human SW1/SNF complex. Nature (1994) 370(6489):477–81. 10.1038/370477a0 8047169

[40] Ho L Crabtree GR. Chromatin remodelling during development. Nature (2010) 463(7280):474–84. 10.1038/nature08911 20110991 PMC3060774

[41] Hargreaves DC Crabtree GR. ATP-dependent chromatin remodeling: genetics, genomics and mechanisms. Cell Res (2011) 21(3):396–420. 10.1038/cr.2011.32 21358755 PMC3110148

[42] Wu JI. Diverse functions of ATP-dependent chromatin remodeling complexes in development and cancer. Chin J Biochem Biophys (2012) 44(1):54–69. 10.1093/abbs/gmr099 22194014

[43] Papamichos-Chronakis M Peterson CL. Chromatin and the genome integrity network. Nat Rev Genet (2013) 14(1):62–75. 10.1038/nrg3345 23247436 PMC3731064

[44] Klochendler-Yeivin A Fiette L Barra J Muchardt C Babinet C Yaniv M. The murine SNF5/INI1 chromatin remodeling factor is essential for embryonic development and tumor suppression. EMBO Rep (2000) 1(6):500–6. 10.1093/embo-reports/kvd129 11263494 PMC1083796

[45] Roberts CW Galusha SA McMenamin ME Fletcher CD Orkin SH. Haploinsufficiency of Snf5 (integrase interactor 1) predisposes to malignant rhabdoid tumors in mice. Proc Natl Acad Sci U S A (2000) 97(25):13796–800. 10.1073/pnas.250492697 11095756 PMC17655

[46] Guidi CJ Sands AT Zambrowicz BP Turner TK Demers DA Webster W Disruption of Ini1 leads to peri-implantation lethality and tumorigenesis in mice. Mol Cell Biol (2001) 21(10):3598–603. 10.1128/MCB.21.10.3598-3603.2001 11313485 PMC100281

[47] Medina PP Romero OA Kohno T Montuenga LM Pio R Yokota J Frequent BRG1/SMARCA4-inactivating mutations in human lung cancer cell lines. Hum Mutat (2008) 29(5):617–22. 10.1002/humu.20730 18386774

[48] Wang X Sansam CG Thom CS Metzger D Evans JA Nguyen PT Oncogenesis caused by loss of the SNF5 tumor suppressor is dependent on activity of BRG1, the ATPase of the SWI/SNF chromatin remodeling complex. Cancer Res (2009) 69(20):8094–101. 10.1158/0008-5472.CAN-09-0733 19789351 PMC2763035

[49] Oike T Ogiwara H Tominaga Y Ito K Ando O Tsuta K A synthetic lethality-based strategy to treat cancers harboring a genetic deficiency in the chromatin remodeling factor BRG1. Cancer Res (2013) 73(17):5508–18. 10.1158/0008-5472.CAN-12-4593 23872584

[50] Hoffman GR Rahal R Buxton F Xiang K McAllister G Frias E Functional epigenetics approach identifies BRM/SMARCA2 as a critical synthetic lethal target in BRG1-deficient cancers. Proc Natl Acad Sci U S A (2014) 111(8):3128–33. 10.1073/pnas.1316793111 24520176 PMC3939885

[51] Sentani K Oue N Kondo H Kuraoka K Motoshita J Ito R Increased expression but not genetic alteration of BRG1, a component of the SWI/SNF complex, is associated with the advanced stage of human gastric carcinomas. Pathobiology (2001) 69(6):315–20. 10.1159/000064638 12324708

[52] Sun A Tawfik O Gayed B Thrasher JB Hoestje S Li C Aberrant expression of SWI/SNF catalytic subunits BRG1/BRM is associated with tumor development and increased invasiveness in prostate cancers. Prostate (2007) 67(2):203–13. 10.1002/pros.20521 17075831

[53] Saladi SV Keenen B Marathe HG Qi H Chin KV de la Serna IL. Modulation of extracellular matrix/adhesion molecule expression by BRG1 is associated with increased melanoma invasiveness. Mol Cancer (2010) 9:280. 10.1186/1476-4598-9-280 20969766 PMC3098014

[54] Watanabe T Semba S Yokozaki H. Regulation of PTEN expression by the SWI/SNF chromatin-remodelling protein BRG1 in human colorectal carcinoma cells. Br J Cancer (2011) 104(1):146–54. 10.1038/sj.bjc.6606018 21102582 PMC3039810

[55] Bai J Mei P Zhang C Chen F Li C Pan Z BRG1 is a prognostic marker and potential therapeutic target in human breast cancer. PLoS One (2013) 8(3):e59772. 10.1371/journal.pone.0059772 23533649 PMC3606107

[56] Shi J Whyte WA Zepeda-Mendoza CJ Milazzo JP Shen C Roe JS Role of SWI/SNF in acute leukemia maintenance and enhancer-mediated Myc regulation. Genes Dev (2013) 27(24):2648–62. 10.1101/gad.232710.113 24285714 PMC3877755

[57] Liu X Tian X Wang F Ma Y Kornmann M Yang Y. BRG1 promotes chemoresistance of pancreatic cancer cells through crosstalking with Akt signalling. Eur J Cancer (2014) 50(13):2251–62. 10.1016/j.ejca.2014.05.017 24953335

[58] Fillmore CM Xu C Desai PT Berry JM Rowbotham SP Lin YJ EZH2 inhibition sensitizes BRG1 and EGFR mutant lung tumours to TopoII inhibitors. Nature (2015) 520(7546):239–42. 10.1038/nature14122 25629630 PMC4393352

[59] Wu Q Madany P Akech J Dobson JR Douthwright S Browne G The SWI/SNF ATPases are required for triple negative breast cancer cell proliferation. J Cell Physiol (2015) 230:2683–94. 10.1002/jcp.24991 25808524 PMC4516601

[60] Jubierre L Soriano A Planells-Ferrer L Paris-Coderch L Tenbaum SP Romero OA BRG1/SMARCA4 is essential for neuroblastoma cell viability through modulation of cell death and survival pathways. Oncogene (2016) 35(39):5179–90. 10.1038/onc.2016.50 26996667

[61] Bultman S Gebuhr T Yee D La Mantia C Nicholson J Gilliam A A Brg1 null mutation in the mouse reveals functional differences among mammalian SWI/SNF complexes. Mol Cell (2000) 6(6):1287–95. 10.1016/S1097-2765(00)00127-1 11163203

[62] Bultman SJ Herschkowitz JI Godfrey V Gebuhr TC Yaniv M Perou CM Characterization of mammary tumors from Brg1 heterozygous mice. Oncogene (2008) 27(4):460–8. 10.1038/sj.onc.1210664 17637742

[63] Serber DW Rogala A Makarem M Rosson GB Simin K Godfrey V The BRG1 chromatin remodeler protects against ovarian cysts, uterine tumors, and mammary tumors in a lineage-specific manner. PLoS One (2012) 7(2):e31346. 10.1371/journal.pone.0031346 22363625 PMC3283619

[64] Network TCGA. Comprehensive molecular portraits of human breast tumours. Nature (2012) 490(7418):61–70. 10.1038/nature11412 23000897 PMC3465532

[65] Menendez JA Lupu R Fatty acid synthase and the lipogenic phenotype in cancer pathogenesis. Nat Rev Cancer (2007) 7(10):763–77. 10.1038/nrc2222 17882277

[66] Vazquez-Martin A Colomer R Brunet J Lupu R Menendez JA. Overexpression of fatty acid synthase gene activates HER1/HER2 tyrosine kinase receptors in human breast epithelial cells. Cell Prolif (2008) 41(1):59–85. 10.1111/j.1365-2184.2007.00498.x 18211286 PMC6496011

[67] Verhoeven G [Androgens and increased lipogenesis in prostate cancer. Cell biologic and clinical perspectives]. Verh K Acad Geneeskd Belg (2002) 64(3):189–195; discussion 195–186. 12238242

[68] Martel PM Bingham CM McGraw CJ Baker CL Morganelli PM Meng ML S14 protein in breast cancer cells: direct evidence of regulation by SREBP-1c, superinduction with progestin, and effects on cell growth. Exp Cell Res (2006) 312(3):278–88. 10.1016/j.yexcr.2005.10.022 16300755

[69] Migita T Narita T Nomura K Miyagi E Inazuka F Matsuura M ATP citrate lyase: activation and therapeutic implications in non-small cell lung cancer. Cancer Res (2008) 68(20):8547–54. 10.1158/0008-5472.CAN-08-1235 18922930

[70] Mukherjee A Wu J Barbour S Fang X. Lysophosphatidic acid activates lipogenic pathways and de novo lipid synthesis in ovarian cancer cells. J Biol Chem (2012) 287(30):24990–5000. 10.1074/jbc.M112.340083 22665482 PMC3408203

[71] Mashima T Seimiya H Tsuruo T. De novo fatty-acid synthesis and related pathways as molecular targets for cancer therapy. Br J Cancer (2009) 100(9):1369–72. 10.1038/sj.bjc.6605007 19352381 PMC2694429

[72] Kuhajda FP Jenner K Wood FD Hennigar RA Jacobs LB Dick JD Fatty acid synthesis: a potential selective target for antineoplastic therapy. Proc Natl Acad Sci U S A (1994) 91(14):6379–83. 10.1073/pnas.91.14.6379 8022791 PMC44205

[73] Zhou W Han WF Landree LE Thupari JN Pinn ML Bililign T Fatty acid synthase inhibition activates AMP-activated protein kinase in SKOV3 human ovarian cancer cells. Cancer Res (2007) 67(7):2964–71. 10.1158/0008-5472.CAN-06-3439 17409402

[74] Kridel SJ Axelrod F Rozenkrantz N Smith JW. Orlistat is a novel inhibitor of fatty acid synthase with antitumor activity. Cancer Res (2004) 64(6):2070–5. 10.1158/0008-5472.CAN-03-3645 15026345

[75] Hatzivassiliou G Zhao F Bauer DE Andreadis C Shaw AN Dhanak D ATP citrate lyase inhibition can suppress tumor cell growth. Cancer Cell (2005) 8(4):311–21. 10.1016/j.ccr.2005.09.008 16226706

[76] Chajes V Cambot M Moreau K Lenoir GM Joulin V. Acetyl-CoA carboxylase alpha is essential to breast cancer cell survival. Cancer Res (2006) 66(10):5287–94. 10.1158/0008-5472.CAN-05-1489 16707454

[77] Zaidi N Swinnen JV Smans K. ATP-citrate lyase: a key player in cancer metabolism. Cancer Res (2012) 72(15):3709–14. 10.1158/0008-5472.CAN-11-4112 22787121

[78] Mertins P Mani DR Ruggles KV Gillette MA Clauser KR Wang P Proteogenomics connects somatic mutations to signalling in breast cancer. Nature (2016) 534(7605):55–62. 10.1038/nature18003 27251275 PMC5102256

[79] Kadoch C Hargreaves DC Hodges C Elias L Ho L Ranish J Proteomic and bioinformatic analysis of mammalian SWI/SNF complexes identifies extensive roles in human malignancy. Nat Genet (2013) 45(6):592–601. 10.1038/ng.2628ng.2628 23644491 PMC3667980

[80] Lin H Wong RP Martinka M Li G. BRG1 expression is increased in human cutaneous melanoma. Br J Dermatol (2010) 163(3):502–10. 10.1111/j.1365-2133.2010.09851.x 20491765

[81] Reisman D Glaros S Thompson EA. The SWI/SNF complex and cancer. Oncogene (2009) 28(14):1653–68. 10.1038/onc.2009.4 19234488

[82] Bourgo RJ Siddiqui H Fox S Solomon D Sansam CG Yaniv M SWI/SNF-deficiency results in aberrant chromatin organization, mitotic failure, and diminished proliferative capacity. Mol Biol Cell (2009) 20(14):3192–9. 10.1091/mbc.E08-12-1224 19458193 PMC2710832

[83] Naidu SR Love IM Imbalzano AN Grossman SR Androphy EJ. The SWI/SNF chromatin remodeling subunit BRG1 is a critical regulator of p53 necessary for proliferation of malignant cells. Oncogene (2009) 28(27):2492–501. 10.1038/onc.2009.121 19448667 PMC2708319

[84] Cohet N Stewart KM Mudhasani R Asirvatham AJ Mallappa C Imbalzano KM SWI/SNF chromatin remodeling enzyme ATPases promote cell proliferation in normal mammary epithelial cells. J Cell Physiol (2010) 223(3):667–78. 10.1002/jcp.22072 20333683 PMC3320666

[85] Wu Q Sharma S Cui H LeBlanc SE Zhang H Muthuswami R Targeting the chromatin remodeling enzyme BRG1 increases the efficacy of chemotherapy drugs in breast cancer cells. Oncotarget (2016) 7:27158–75. 10.18632/oncotarget.8384 27029062 PMC5053639

[86] Fedorov O Castex J Tallant C Owen DR Martin S Aldeghi M Selective targeting of the BRG/PB1 bromodomains impairs embryonic and trophoblast stem cell maintenance. Sci Adv (2015) 1(10):e1500723. 10.1126/sciadv.1500723 26702435 PMC4681344

[87] Vangamudi B Paul TA Shah PK Kost-Alimova M Nottebaum L Shi X The SMARCA2/4 ATPase domain surpasses the bromodomain as a drug target in SWI/SNF mutant cancers: insights from cDNA rescue and PFI-3 inhibitor studies. Cancer Res (2015) 75(18):3865–78. 10.1158/0008-5472.CAN-14-3798 26139243 PMC4755107

[88] Muthuswami R Mesner LD Wang D Hill DA Imbalzano AN Hockensmith JW. Phosphoaminoglycosides inhibit SWI2/SNF2 family DNA-dependent molecular motor domains. Biochemistry (2000) 39(15):4358–65. 10.1021/bi992503r 10757984

[89] Dutta P Tanti GK Sharma S Goswami SK Komath SS Mayo MW Global epigenetic changes induced by SWI2/SNF2 inhibitors characterize neomycin-resistant mammalian cells. PLoS One (2012) 7(11):e49822. 10.1371/journal.pone.0049822 23209606 PMC3509132

[90] Donohoe DR Bultman SJ. Metaboloepigenetics: interrelationships between energy metabolism and epigenetic control of gene expression. J Cell Physiol (2012) 227(9):3169–77. 10.1002/jcp.24054 22261928 PMC3338882

[91] Lu C Thompson CB. Metabolic regulation of epigenetics. Cell Metab (2012) 16(1):9–17. 10.1016/j.cmet.2012.06.001 22768835 PMC3392647

[92] Kaelin WG Jr McKnight SL Influence of metabolism on epigenetics and disease. Cell (2013) 153(1):56–69. 10.1016/j.cell.2013.03.004 23540690 PMC3775362

[93] Wellen KE Hatzivassiliou G Sachdeva UM Bui TV Cross JR Thompson CB. ATP-citrate lyase links cellular metabolism to histone acetylation. Science (2009) 324(5930):1076–80. 10.1126/science.1164097 19461003 PMC2746744

[94] Lin H Su X He B. Protein lysine acylation and cysteine succination by intermediates of energy metabolism. ACS Chem Biol (2012) 7(6):947–60. 10.1021/cb3001793 22571489 PMC3376250

[95] Shi L Tu BP. Acetyl-CoA and the regulation of metabolism: mechanisms and consequences. Curr Opin Cell Biol (2015) 33:125–31. 10.1016/j.ceb.2015.02.003 25703630 PMC4380630

[96] Sabari BR Zhang D Allis CD Zhao Y Metabolic regulation of gene expression through histone acylations. Nat Rev Mol Cell Biol (2016) 18:90–101. 10.1038/nrm.2016.140 27924077 PMC5320945

[97] Liu Z Yang T Li X Peng T Hang HC Li XD Integrative chemical biology approaches for identification and characterization of “erasers” for fatty-acid-acylated lysine residues within proteins. Angew Chem Int Ed Engl (2015) 54(4):1149–52. 10.1002/anie.201408763 25476551 PMC4382910

[98] Imai S Guarente L. NAD+ and sirtuins in aging and disease. Trends Cell Biol (2014) 24(8):464–71. 10.1016/j.tcb.2014.04.002 24786309 PMC4112140

[99] Imai S Armstrong CM Kaeberlein M Guarente L. Transcriptional silencing and longevity protein Sir2 is an NAD-dependent histone deacetylase. Nature (2000) 403(6771):795–800. 10.1038/35001622 10693811

[100] Landry J Slama JT Sternglanz R. Role of NAD(+) in the deacetylase activity of the SIR2-like proteins. Biochem Biophys Res Commun (2000) 278(3):685–90. 10.1006/bbrc.2000.3854 11095969

[101] Smith JS Brachmann CB Celic I Kenna MA Muhammad S Starai VJ A phylogenetically conserved NAD+-dependent protein deacetylase activity in the Sir2 protein family. Proc Natl Acad Sci U S A (2000) 97(12):6658–63. 10.1073/pnas.97.12.6658 10841563 PMC18692

[102] Michishita E Park JY Burneskis JM Barrett JC Horikawa I. Evolutionarily conserved and nonconserved cellular localizations and functions of human SIRT proteins. Mol Biol Cell (2005) 16(10):4623–35. 10.1091/mbc.E05-01-0033 16079181 PMC1237069

[103] Houtkooper RH Canto C Wanders RJ Auwerx J. The secret life of NAD+: an old metabolite controlling new metabolic signaling pathways. Endocr Rev (2010) 31(2):194–223. 10.1210/er.2009-0026 20007326 PMC2852209

[104] Ramsey KM Yoshino J Brace CS Abrassart D Kobayashi Y Marcheva B Circadian clock feedback cycle through NAMPT-mediated NAD+ biosynthesis. Science (2009) 324(5927):651–4. 10.1126/science.1171641 19299583 PMC2738420

[105] Koltai E Szabo Z Atalay M Boldogh I Naito H Goto S Exercise alters SIRT1, SIRT6, NAD and NAMPT levels in skeletal muscle of aged rats. Mech Ageing Dev (2010) 131(1):21–8. 10.1016/j.mad.2009.11.002 19913571 PMC2872991

[106] Fulco M Cen Y Zhao P Hoffman EP McBurney MW Sauve AA Glucose restriction inhibits skeletal myoblast differentiation by activating SIRT1 through AMPK-mediated regulation of Nampt. Dev Cell (2008) 14(5):661–73. 10.1016/j.devcel.2008.02.004 18477450 PMC2431467

[107] Rodgers JT Lerin C Haas W Gygi SP Spiegelman BM Puigserver P. Nutrient control of glucose homeostasis through a complex of PGC-1alpha and SIRT1. Nature (2005) 434(7029):113–8. 10.1038/nature03354 15744310

[108] Canto C Gerhart-Hines Z Feige JN Lagouge M Noriega L Milne JC AMPK regulates energy expenditure by modulating NAD+ metabolism and SIRT1 activity. Nature (2009) 458(7241):1056–60. 10.1038/nature07813 19262508 PMC3616311

[109] Luu TH Morgan RJ Leong L Lim D McNamara M Portnow J A phase II trial of vorinostat (suberoylanilide hydroxamic acid) in metastatic breast cancer: a California Cancer Consortium study. Clin Cancer Res (2008) 14(21):7138–42. 10.1158/1078-0432.CCR-08-0122 18981013 PMC3543872

[110] Ramaswamy B Fiskus W Cohen B Pellegrino C Hershman DL Chuang E Phase I-II study of vorinostat plus paclitaxel and bevacizumab in metastatic breast cancer: evidence for vorinostat-induced tubulin acetylation and Hsp90 inhibition in vivo. Breast Cancer Res Treat (2012) 132(3):1063–72. 10.1007/s10549-011-1928-x 22200869 PMC3486521

[111] Stearns V Jacobs LK Fackler M Tsangaris TN Rudek MA Higgins M Biomarker modulation following short-term vorinostat in women with newly diagnosed primary breast cancer. Clin Cancer Res (2013) 19(14):4008–16. 10.1158/1078-0432.CCR-13-0033 23719261 PMC3718062

[112] Yardley DA Ismail-Khan RR Melichar B Lichinitser M Munster PN Klein PM Randomized phase II, double-blind, placebo-controlled study of exemestane with or without entinostat in postmenopausal women with locally recurrent or metastatic estrogen receptor-positive breast cancer progressing on treatment with a nonsteroidal aromatase inhibitor. J Clin Oncol (2013) 31(17):2128–35. 10.1200/JCO.2012.43.7251 23650416 PMC4881332

[113] Munster P Marchion D Bicaku E Lacevic M Kim J Centeno B Clinical and biological effects of valproic acid as a histone deacetylase inhibitor on tumor and surrogate tissues: phase I/II trial of valproic acid and epirubicin/FEC. Clin Cancer Res (2009) 15(7):2488–96. 10.1158/1078-0432.CCR-08-1930 19318486

[114] Robertson FM Chu K Boley KM Ye Z Liu H Wright MC The class I HDAC inhibitor romidepsin targets inflammatory breast cancer tumor emboli and synergizes with paclitaxel to inhibit metastasis. J Exp Ther Oncol (2013) 10(3):219–33. 24416998

[115] Duvic M Talpur R Ni X Zhang C Hazarika P Kelly C Phase 2 trial of oral vorinostat (suberoylanilide hydroxamic acid, SAHA) for refractory cutaneous T-cell lymphoma (CTCL). Blood (2007) 109(1):31–9. 10.1182/blood-2006-06-025999 16960145 PMC1785068

[116] Olsen EA Kim YH Kuzel TM Pacheco TR Foss FM Parker S Phase IIb multicenter trial of vorinostat in patients with persistent, progressive, or treatment refractory cutaneous T-cell lymphoma. J Clin Oncol (2007) 25(21):3109–15. 10.1200/JCO.2006.10.2434 17577020

[117] Fenaux P Mufti GJ Hellstrom-Lindberg E Santini V Finelli C Giagounidis A Efficacy of azacitidine compared with that of conventional care regimens in the treatment of higher-risk myelodysplastic syndromes: a randomised, open-label, phase III study. Lancet Oncol (2009) 10(3):223–32. 10.1016/S1470-2045(09)70003-8 19230772 PMC4086808

[118] Fenaux P Mufti GJ Hellstrom-Lindberg E Santini V Gattermann N Germing U Azacitidine prolongs overall survival compared with conventional care regimens in elderly patients with low bone marrow blast count acute myeloid leukemia. J Clin Oncol (2010) 28(4):562–9. 10.1200/JCO.2009.23.8329 20026804

[119] Borges S Doppler H Perez EA Andorfer CA Sun Z Anastasiadis PZ Pharmacologic reversion of epigenetic silencing of the PRKD1 promoter blocks breast tumor cell invasion and metastasis. Breast Cancer Res (2013) 15(2):R66. 10.1186/bcr3460 23971832 PMC4052945

[120] Borges S Doppler HR Storz P. A combination treatment with DNA methyltransferase inhibitors and suramin decreases invasiveness of breast cancer cells. Breast Cancer Res Treat (2014) 144(1):79–91. 10.1007/s10549-014-2857-2 24510012 PMC3982927

